# How to Lend Money

**Published:** 1878-04

**Authors:** 


### HOW TO LEND MONEY,

### IF YOU LEND AT ALL:

*To your friends!* As a pure business transaction, **you may** not be too careful. But when a friend of other years comes along, who has not been as successful as yourself, whom disappointment or misplaced confidence, or unavoidable calamity has pressed to the earth, a friend who was once your equal in all things, inferior in none, except perhaps in that hardness of character, which is a general element of success in life, don't begin to hem and haw, and stroke your chin; don't talk about "*buts*" and "*whys*," and the "*tightness of the money market*," he knows that already—spare him the intelligence that you "once loaned Mr. so and so a sum of money, which was never returned;" he don't want your biography, he wants your cash. Don't remind him that if he were to die, you would lose it; that arrow may sink deeper into his heart than any amount of money could ever fathom, and then, close with a recital of this, that and the other thing, which, if really true, could not materially interfere with your furnishing him the required amount. If you have ordinary sagacity, you can make up your mind in a moment, whether to grant the accommodation or to refuse it. If you are a man and you design a refusal, tell him at once in some kindly way, that you do not feel prepared to accede to his wishes. If on the other hand, you have a heart to help him, don't do it as if you felt it were a mountain grinding you to powder, or as if each dollar you parted from, was inflicting a pain equal to the drawing of a tooth; don't torture him with cross questioning, nor worm out of him some of the most sacred secrets of his life; away with your inquisitorial, brassy impertinence; don't lay him on the rack for an hour at a time, as if you gloated at the sacrifice of his manhood, as if you wished to make him go down on his very knees to win his way into your purse, away with it all we say, and stand up like a man; give him a cordial greeting, let a holy sunshine light up your countenance, and speak out before he has done asking, tell him how much you are gratified at having it in your power to help him, and let that help go out in a full, free soul, and with a good slap on the shoulder, bid him look upward and ahead for there's sunshine there for him. Why the very feeling in that man's heart as he goes away from you, is worth more to humanity, than all the money you let him have, ten times told. He goes out of your presence with a heart as light as a feather, in love with all the world, and full of admiring gratitude towards you. He feels his manhood, he feels that confidence is reposed in him, that he is still a man, and this conviction nerves him up to a resolution, to an ambition, to an energy which are of themselves a guarantee of after success. He goes to work with a will, which hews down the obstacles and melts away the ice-bergs which hedge up the ways of men, and behold in a moment, rough places are made smooth, and straight places made plain to him.

Reader! suppose you never get your money back, and you have a heart so big, that you can, notwithstanding his non-payment, give him at every meeting a cordial smile of friendly recognition, can speak to him without ever reminding him of his indebtedness; it may be that you are his only friend, but then you are the world to him, and however hardly that world may have dealt with him, your single exception is placed to the credit side of humanity, a thousand times its individual value; that man can never die a misanthrope, for he will insist upon it to his latest breath, "there's kindness in the world after all."—What a grand thing it is to have a man close his eyes in death, and one of the last thoughts of mortality be a prayer for blessings on your head.

We repeat, then, if you lend money at all, do so freely, promptly, do it with a whole soul. Do it with a grace that becomes a man, with a cordiality which will do quite as much as your money in raising your friend from the depressing influences which surround him. We do not advise the loan of money in any given case, but write to show in what manner it should be done, when decided upon, to bring the most pleasant reminiscences to yourself hereafter, and to carry with it the largest advantages to him whom you wish to befriend.

